# Thermal Conductivity of Liquid *trans*-1,2-Dichloroethene (R-1130(E)): Measurement and Modeling

**DOI:** 10.1007/s10765-024-03334-2

**Published:** 2024

**Authors:** Karim S. Al-Barghouti, Aaron J. Rowane, Ian H. Bell, Marcia L. Huber, Richard A. Perkins

**Affiliations:** 1 Department of Chemical and Petroleum Engineering, University of Kansas, Lawrence, KS 66045, USA; 2 Material Measurement Laboratory, Applied Chemicals and Materials Division, National Institute of Standards and Technology, Boulder, CO 80305, USA

**Keywords:** Extended corresponding states, Low GWP, Residual entropy scaling, R-1130(E), Thermal Conductivity, *Trans*-1,2-dichloroethene, Transient hot-wire

## Abstract

The thermal conductivity of liquid *trans*-1,2-dichloroethene (R-1130(E)) was measured at temperatures ranging from 240 K to 340 K and pressures up to 25 MPa using a transient hot-wire instrument. A total of 447 thermal conductivity data points were measured along six isotherms. Each isotherm includes data at nine pressures, which were chosen to be at equal density increments starting at a pressure of 0.1 MPa (or slightly above the saturation pressure of R-1130(E) at temperatures above its normal boiling point) to a maximum pressure of 25 MPa. The combined expanded uncertainty of the presented experimental data is 1.4% at a 95% confidence level. The experimental data were used to evaluate the performance of an extended corresponding states (ECS) model and a residual entropy scaling (RES) model. Both models were applied in a totally predictive mode, and in a mode where the experimental data were used to tune the model parameters. A volume-translated Peng–Robinson equation of state was used to provide thermodynamic properties needed to apply both models. In a totally predictive mode, the ECS model had an average absolute relative deviation (Δ_AARD_) of 6.89% relative to the experimental data with the largest deviation being − 8.33%. The RES model in a totally predictive mode showed an Δ_AARD_ of 2.55% with the largest deviation being − 5.81%. When model parameters were fitted to the experimental data, both the ECS and the RES model represented the experimental data to within its uncertainty of 1.4%.

## 1 Introduction

In accordance with extensive environmental legislation, 2,2-dichloro-1,1,1-trifluoroethane (R-123; CAS no. 306-83-2) is being phased out of use in the refrigeration sector [1–6]. One proposed replacement for R-123 in low-pressure centrifugal chillers is a refrigerant blend with the ASHRAE designation R-514A [7] owing to its low global warming potential (GWP = 2) [8] and negligible ozone depletion potential. R-514A is an azeotropic mixture consisting of 25.3% mass of the hydrochloroolefin (HCO) *trans*-1,2-dichloroethene (R-1130(E); CAS no. 156-60-5) and 74.7% mass of the hydrofluoroolefin (HFO) *cis*-1,1,1,4,4,4-hexaflouro-2-butene (R-1336mzz(Z); CAS no. 692-49-9). R-514A has an ASHRAE safety classification of B1 meaning it has no flame propagation but a “higher” toxicity [7].

Proper assessment of feasibility, system retrofitting, and assessment of optimal design in associated refrigeration equipment requires proper knowledge of the thermophysical properties of the employed refrigerant(s). R-1336mzz(Z) has been proposed in various applications and relatively abundant information on its thermophysical properties is available [9–13]. In contrast, only a few studies report limited data on the thermophysical properties of R-1130(E). Awbery and Griffiths [14] measured the viscosity of liquid R-1130(E) at temperatures ranging from 258.15 K to 303.15 K using a falling-plug viscometer. Their sample initially included over 10% of *cis*-1,2-dichloroethene and was further purified to an unspecified extent. Ketelaar et al. [15] utilized an Ostwald viscometer to measure the viscosity of liquid R-1130(E) at temperatures ranging from 213.15 K to 298.15 K. Ketelaar and coworkers [15] used rigorous techniques to purify their sample and assessed the degree of purity using dielectric constant measurements but did not explicitly quantify their sample purity. A study by Bates and coworkers [16] employing a form of a guarded plate method reports three thermal conductivity data points of liquid R-1130(E) at (293.15, 303.15, 313.1) K with no specified sample purity. Straka and coworkers [17] measured the isobaric heat capacities of liquid R-1130(E) at temperatures from 268.30 K to 309.14 K with a reported mole fraction purity of 0.997. Most recently, Tanaka and coworkers [18] used an isochoric method to measure densities of R-1130(E) with a reported purity of > 0.997 mass fraction at temperatures ranging from 329 K to 453 K. The same group measured the surface tension of R-1130(E) with a differential capillary rise method between 228 K and 373 K. Several earlier studies report liquid densities of R-1130(E) at ambient pressure and a range of temperatures (288.64 K to 313.15 K) [19–22].

Here, we employ a transient hot-wire (THW) technique to measure the thermal conductivity of R-1130(E) at temperatures ranging from 240 K to 340 K and pressures up to 25 MPa. The experimental thermal conductivity data presented here are used to assess approximation capabilities of an extended corresponding states (ECS) model [23–25] and a previously proposed residual entropy scaling (RES) model [26]. The measured data are used to tune both RES and ECS model parameters to accurately evaluate the thermal conductivity over a wide range of conditions.

## 2 Materials and Methods

### 2.1 Materials

R-1130(E) with CAS no. 156-60-5 and molar mass of 96.938 g·mol^−1^ [27] was obtained from The Chemours Company^1^ with an assessed purity of 0.991 mass fraction. The purity of the sample was assessed using ^1^H NMR as described in the supplementary information (SI). The sample was transferred to a stainless-steel cylinder then degassed using the freeze/pump/thaw technique, which involves freezing the sample with liquid nitrogen then pumping out the vapor space to remove volatile impurities such as air. The “thaw” step in this process involved heating the sample to drive any remaining volatile impurities to the sample vapor space. This process was repeated until the pressure over the frozen sample was less than 0.01 Pa. The freeze/pump/thaw technique is described in more detail elsewhere [28]. Sample specifications are listed in Table 1.

### 2.2 Transient Hot-Wire: Measurement Principle

The THW technique relates the thermal conductivity of a fluid to the temperature rise of a wire induced by an applied power over a given short duration. The THW technique uses the slope of a linear fit to the ideal temperature rise of the wire versus natural logarithm of time. The governing equation for the THW method is

(1) $$\Delta T_{\text{id}}=\Delta T_{\text{W}}+\sum_{i=1}\delta T_{i}=\frac{q}{4\pi \lambda }\left[\ln(t)+\ln\left(\frac{4\alpha }{r_{0}^{2}C}\right)\right],$$

where $\Delta T_{\text{id}}$ is the ideal temperature rise of the wire, $\Delta T_{W}$ is the measured temperature rise of the wire, $\sum \delta T_{i}$ are the set of necessary corrections, q is the applied power per unit length, λ is the thermal conductivity, t is the time, $r_{0}$ is the radius of the wire, α is the thermal diffusivity of the fluid, and C is the exponential of Euler’s constant (C=1.781…). Corrections to the observed temperature rise are required to account for deviations from ideal-line-source conduction. A comprehensive assessment of required corrections is detailed by Healy and coworkers [29].

### 2.3 Transient Hot-Wire: Instrumentation

The utilized THW instrument has been described in previous work [30]. The instrument employs two bare platinum hot-wires with a diameter of 12.7 μm each. The dual-wire technique compensates for axial conduction due to temperature gradients near the wire end supports. The platinum wires serve as both resistance thermometers and as electric heat sources. The dual-wire assembly is situated inside a copper pressure vessel, which is located inside a cryostat. The apparatus can operate at pressures up to 70 MPa and temperatures from 60 K to 345 K. However, in this study the temperature range was limited to 240 K to 340 K to avoid solidification of R-1130(E), which has a triple point of 223.71 K [18]. The cell was connected to a manifold located outside of the cryostat that remained at room temperature. Connected to the manifold is a sample filling line, sample venting line, a pressure transducer, and a hand pump that also remains at room temperature. The pressure transducer uncertainty was 7 kPa with a maximum pressure of 70 MPa. The hand pump was used to increase/decrease the system pressure. The cell temperature was monitored using a standard platinum resistance thermometer (SPRT) calibrated to an uncertainty of 0.005 K.

### 2.4 Measurement Principle

The thermal conductivity apparatus including the tubing, cell, pressure transducer, and hand pump were evacuated prior to sample filling. The sample was loaded into the thermal conductivity cell by cooling the cell to ~ 240 K and gradually heating an inverted sample cylinder using an infrared heating lamp. A capillary heater was used to prevent solidification of the sample in the tubes. A temperature rise versus time test run was performed to verify that the cell was filled with liquid sample. The sample was also allowed to fill the tubing, pressure transducer, and hand pump, which remained at room temperature.

Measurements were conducted at five power levels corresponding to varying temperature rises. Drive voltages were selected such that the resulting temperature rise was between 2 K and 4 K. Temperature rises within this range provided sufficient resolution for quality measurements with reduced uncertainty and avoided excessively high temperature rises, which would induce convection. All measurements were performed at time intervals of 1.0 s or less to avoid fluid convection. To avoid/assess any deviations induced by the potential presence of ionic impurities, measurements with an alternating current (AC) power source at 1000 Hz [31] were conducted in tandem with measurements employing a direct current (DC) power source. No noticeable differences were seen between measurements using AC and DC power sources. The thermal conductivity of R-1130(E) is reported here at temperatures ranging between 240 K and 340 K at increments of 20 K and pressures up to 25 MPa. Nine pressure points were chosen for each isotherm based on equal density increments approximated using a volume-translated Peng–Robinson EoS [32–34] fit to the data of Tanaka et al. [18] as described in Sect. 3.2.1. The data reported here encompass measurement repeats at similar state points including varying power inputs and different electrical currents (AC and DC). Each thermal conductivity data point is reported at the average wire temperature, $T_{e}$, over the span of the elapsed time of measurement as given by

(2) $$T_{e}=\frac{T_{2}\;+\;T_{1}}{2},$$

where $T_{1}$ and $T_{2}$ correspond to the wire temperatures at the onset of and end of measurement, respectively. The average deviation from the slope of the ideal-line source temperature rise versus ln(t) was 1.3%. Accounting for the uncertainties in pressure measurements, temperature measurements of the cell and wire, power input per unit length of wire, estimated density, and the deviation of the line fit to the ideal line, the average combined expanded uncertainty in the reported thermal conductivity values was 1.4% with a coverage factor, k=2. The average combined expanded uncertainty was evaluated using the propagation of error formulation found in the *Guidelines for Evaluating and Expressing the Uncertainty of NIST Measurement Results* [35]. More information on the respective contributions and calculations of uncertainty is provided in the SI.

## 3 Experimental Results

### 3.1 Experimental Thermal Conductivity

**Fig. 1:**
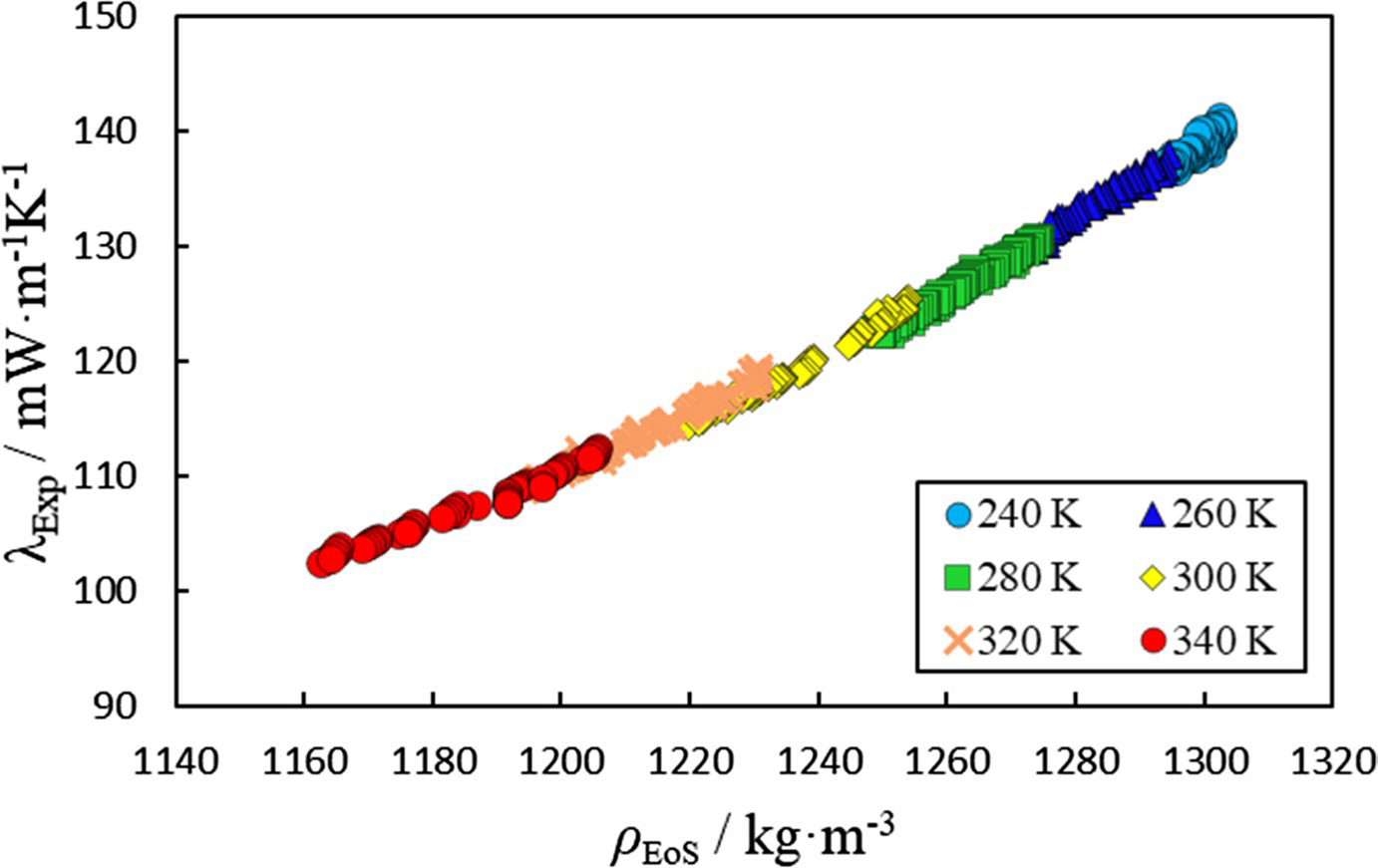
Measured thermal conductivity of R-1130(E) ($\lambda_{\text{Exp}}$) as a function of density ($\rho_{\text{EoS}}$) at six nominal temperatures. $\rho_{\text{EoS}}$ are densities calculated using a volume-translated Peng–Robinson EoS fitted to experimental liquid-phase density data reported by Tanaka et al. [18]

The measured thermal conductivity at each measurement condition with the respective cell temperature, wire temperature, power input to wire per unit length, density, input power frequency, and deviation from the ideal ΔT versus ln(t) line (STAT) are presented as a separate data file in the SI. The experimental data can also be found at data.nist.gov (https://doi.org/https://doi.org/10.18434/mds2-3108). Figure 1 illustrates the measured thermal conductivity as a function of density at several nominal temperatures. The nominal temperatures presented in Fig. 1 are those associated with the approximate cell temperature (the actual experimental temperatures vary depending on the induced temperature rise of the wire). The EoS represents the liquid density within 0.3% relative to the reported experimental data. The details of the adopted model are presented in Sect. 3.2.

The data presented in this study are interpolated to the temperatures and pressure of the three state points of which Bates and coworkers [16] present their measured data in 1941. The Δ_AARD_ between the data presented in this study and those of Bates and coworkers is 6.61%, with the deviations being consistently negative as illustrated in the SI. Further details on the interpolation and comparison are presented in the SI.

### 3.2 Modeling Framework

In this work, we test the performance of an extended corresponding states (ECS) model and a residual entropy scaling (RES) model to evaluate the thermal conductivity at the conditions investigated. Both the ECS and RES models are tested in predictive and correlative modes. Thermodynamic properties needed to utilize both the ECS and RES models are estimated using a volume-translated Peng–Robinson EoS [32–34] fitted to published experimental density data [18]. The performance of each model is characterized by the average absolute relative deviation (Δ_AARD_),

(3) $$\Delta_{\text{AARD}}=\frac{100}{N}\sum_{i=1}^{N}\left|\frac{x_{\mathrm{Exp}}-x_{\text{Model}}}{x_{\mathrm{Exp}}}\right|,$$

where $x_{\text{Exp}}$ is an experimental thermophysical property and $x_{\text{Model}}$ is a value calculated with either the ECS or RES model. The maximum deviation is calculated as

(4) $$\Delta_{max}=MAX\left(100\cdot \left|\frac{x_{Exp}-x_{\text{Model}}}{x_{Exp}}\right|\right).$$

### 3.3 Thermodynamic Modeling

It is first necessary to have an EoS for the thermodynamic properties of R-1130(E) as mentioned. Teraishi et al. [36] developed an extended corresponding states (ECS) model for HFO refrigerants incorporating universal parameters that depend only on the critical parameters $T_{c},\;p_{c},\;\rho_{c}$, and the acentric factor, ω. Tanaka et al. [18] applied this model to R-1130(E) and made comparisons with their experimental density data. These comparisons indicated that the ECS model represented the liquid-phase density data to within 1% with an average deviation of 0.55% with a systematic negative deviation. This is significantly larger than the experimental uncertainty, which is estimated by Tanaka et el. [18] to be 0.3% (at the 95% confidence level).

#### 3.3.1 Volume-Translated Peng–Robinson Equation of State

Due to the paucity of experimental data, it is premature to develop a Helmholtz energy EoS as this type of equation involves simultaneously fitting multiple thermodynamic properties including speed of sound, density, and vapor pressure. In an effort to obtain a better representation of the liquid density than what was presented in Tanaka et al. [18], we used the relatively simple volume-translated Peng–Robinson [32, 33] (vtPR) EoS. The volume translation concept was introduced by Peneloux et al. [33]. However, we used the implementation described in Diky et al. [34], where the translation is considered a constant. When the translation parameter is set to zero, the original Peng–Robinson [32] EoS is recovered. We fitted the liquid-phase density data of Tanaka et al. [18] to the vtPR [32, 33] incorporating the same $T_{c},\;p_{c},\;\rho_{c}$, and acentric factor, ω, as were used by Tanaka et al. [18], namely $T_{c}=516.5\;K,\;p_{c}=5.510\;MPa,\;\rho_{c}=4.435\;mol\cdot {dm}^{-3}$, and ω=0.2137. We also used the same expression for the ideal-gas heat capacity as in Tanaka et al. [18]. Upon fitting the liquid density data of Tanaka et al. [18], we obtained a translation parameter value of −0.00578 m^3^/kmol.

Comparisons of the results of the vtPR equation and the ECS model of Teraishi et al. [36] with the experimental data of Tanaka et al. [18] are presented in Figures S1–S6 of the SI. Figures S1 and S2 show comparisons with the vtPR and ECS models for the experimental density data of Tanaka et al. [18] in the liquid phase. As discussed earlier, the estimated uncertainty of the experimental data is 0.3% at the 95% confidence level. Figure S1 shows that the data are represented to within this level of uncertainty and systematic deviations are not observed for the vtPR model. This is a slight improvement over the ECS model used by Tanaka et al. [18] shown in Figure S2, but is still much higher than the uncertainty level expected of many other low-GWP refrigerants that incorporate Helmholtz equations of state. For example, the recent Helmholtz EoS for R-1234yf by Lemmon and Akasaka [37] has an uncertainty in liquid density of 0.1%. The vtPR model implemented here for R-1130(E) is included in the SI as a fluid file compatible with REFPROP v10 [38].

#### 3.3.2 Transport Property ECS Model: Thermal Conductivity

**Fig. 2:**
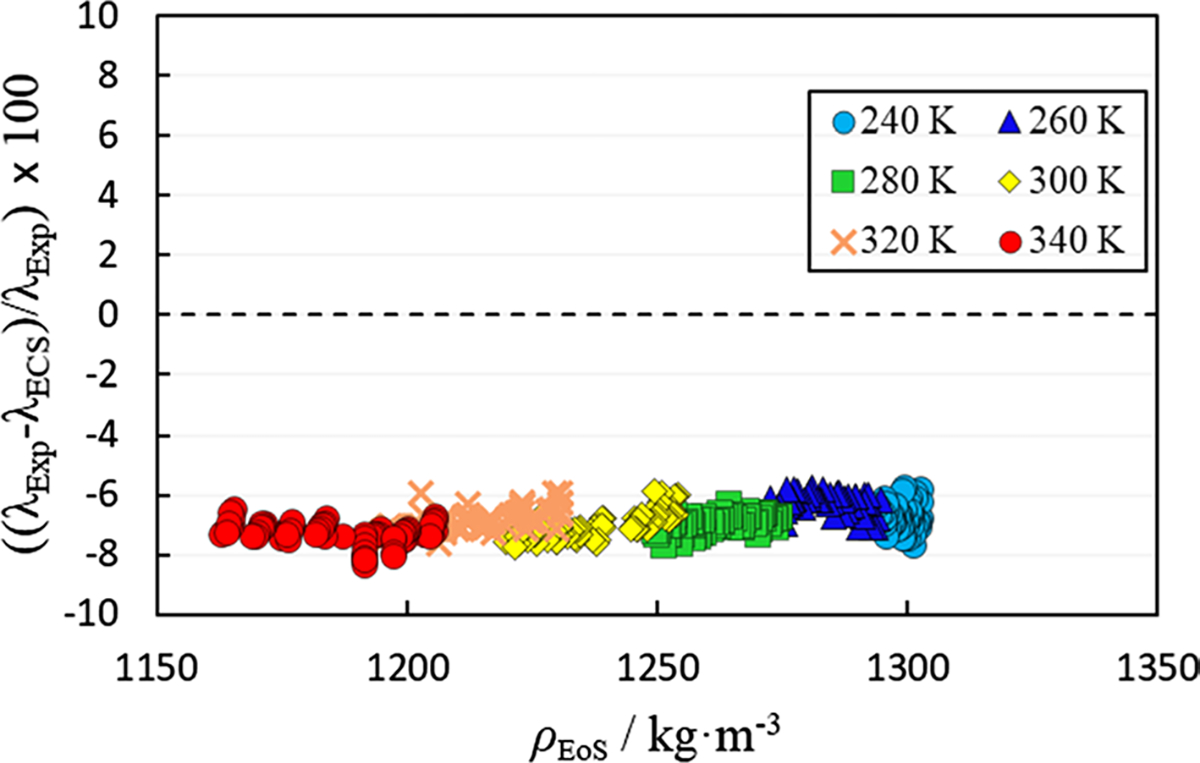
Comparison of experimentally measured thermal conductivity values ($\lambda_{\text{Exp}}$) to those predicted using the extended corresponding states model in a predictive mode ($\lambda_{ECS}$) as a function of density ($\rho_{\text{EoS}}).\;\rho_{\text{EoS}}$ is the density calculated using the volume-translated Peng–Robinson EoS described in Sect. 3.2.1. Temperatures in legend are nominal temperatures

The extended corresponding states model for transport properties has been described previously [23–25] and will only briefly be discussed here. Implementation of the ECS models requires a Lennard–Jones collision diameter, σ, and a Lennard–Jones pair potential well depth, ε, which were estimated using the method of Chung et al. [39] using the critical point values used in Tanaka et al. [18], resulting in σ=0.492nm and $\varepsilon /\left(k_{B}\right)=410.15\;K$, where $k_{B}$ is the Boltzmann constant. The ECS model as described in Huber [23] was first used in a predictive approach with no fitted coefficients and was then improved by fitting the thermal conductivity data measured in this work. The resulting coefficients to Eq. 21 in Huber [23],

(5) $$\chi \left(\rho_{\text{r}}\right)=\sum_{k=0}^{n}b_{k}\rho_{\text{r}}^{k},$$

were found to be $b_{0}=0.911215$ and $b_{1}=-0.0217939$ using a linear fit in reduced density. Due to the lack of dilute-gas thermal conductivity data for R-1130(E), the dilute-gas thermal conductivity coefficient, $f_{\text{int}}$ (defined in Eq. 16 in Ref. [23]), was set to the constant value 0.00125 W·m^−1^·K^−1^. The critical enhancement of thermal conductivity was estimated with the method of Perkins et al. [40]. Figure 2 illustrates the deviations of the predictive ECS model approach compared to the experimental thermal conductivity measurements at varying densities (densities calculated from the vtPR EoS). The Δ_AARD_ of the predictive approach is 6.89%. The nominal temperatures presented in Fig. 2 and the subsequent deviation graphs are those associated with the approximate cell temperature (the actual experimental temperatures varied depending on the induced temperature rise of the wire).

**Fig. 3:**
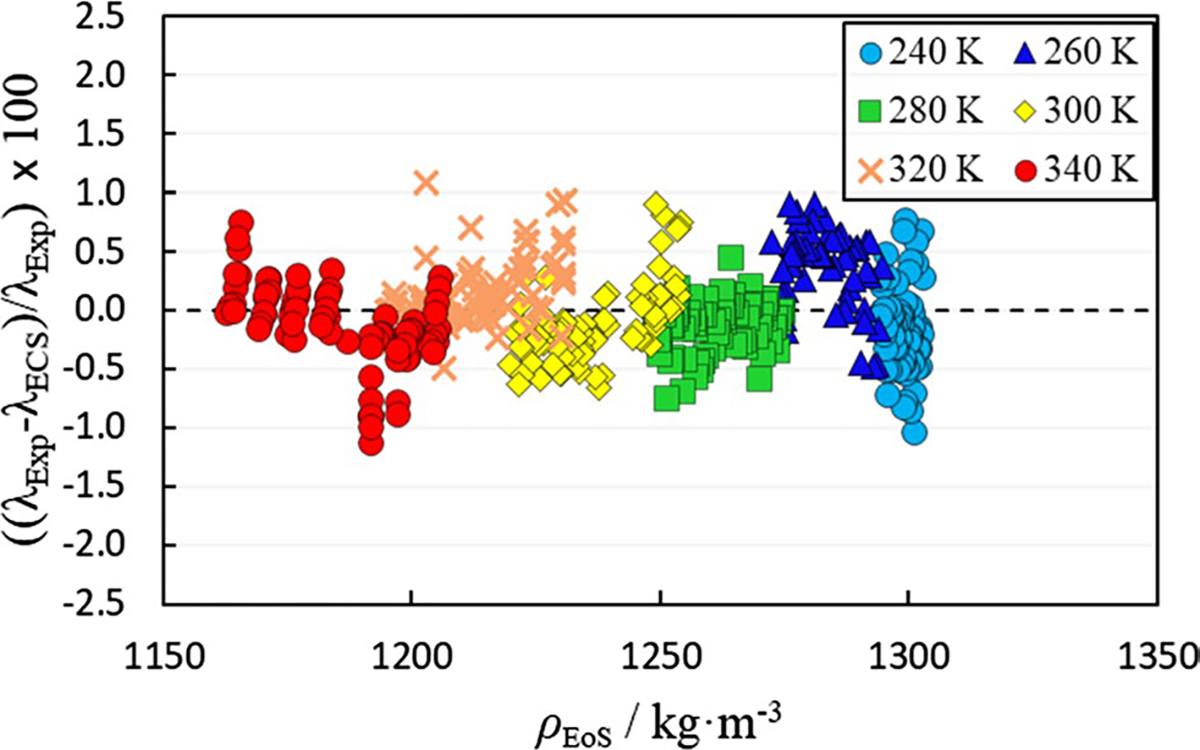
Comparison of experimentally measured thermal conductivity values ($\lambda_{\text{Exp}}$) to those calculated using the extended corresponding states model with fitted parameters ($\lambda_{ECS}$) as a function of density ($\rho_{\text{EoS}}).\;\rho_{\text{EoS}}$ is the density calculated using the volume-translated Peng–Robinson EoS described in Sect. 3.2.1. Temperatures in legend are nominal temperatures

Figure 3 shows a comparison of the thermal conductivity calculated using the ECS model employing parameters fitted to the data and the experimental data at varying densities calculated from the vtPR EoS. The Δ_AARD_ of the ECS model with regressed coefficients is 0.28% relative to the experimental data and there is no systematic bias. The SI contains an ECS model file that can be used with REFPROP v10. The provided ECS model file can also approximate the viscosity of the fluid as described in the SI.

#### 3.3.3 Residual Entropy Scaling

The residual entropy scaling model developed by Yang and coworkers [26] is used to calculate the thermal conductivity of the pure fluid. An overview of the approach follows and further details can be found in the work of Yang et al. [26]

The molar residual entropy, $s_{\text{res}}$, can be defined as

(6) $$s_{res}=s\left(T,\rho \right)-s_{ig}\left(T,\rho \right),$$

in which s(T,ρ) is the entropy at the temperature and molar density of interest and $s_{ig}(T,\rho )$ is the ideal gas entropy at the same temperature and molar density. A dimensionless residual entropy, $s^{+}$, is defined as

(7) $$s^{+}=-s_{res}/R,$$

where R is the universal gas constant in J mol^−1^ K^−1^. The residual entropy, and thus the dimensionless residual entropy, is calculated using the vtPR EoS model described above and implemented as a fluid file in REFPROP [36, 38]. The Rosenfeld-scaled [41] thermal conductivity, $\tilde{\lambda }$, is then defined as [42]

(8) $$\tilde{\lambda }=\frac{\lambda }{k_{B}\rho_{N}^{2/3}\sqrt{k_{B}T/m}}.$$

Here, $\tilde{\lambda }$ is the thermal conductivity scaled by the length, time, and energy dimensions. $k_{B}$ is the Boltzmann constant in J K^−1^, $\rho_{N}$ is the number density in m^−3^, and m is the mass of one molecule in kg.

The plus-scaled thermal conductivity proposed by Bell and coworkers [42, 43] is

(9) $$\lambda^{+}=\tilde{\lambda }\times {\left(s^{+}\right)}^{2/3},$$

yielding a dimensionless residual thermal conductivity defined by

(10) $$\lambda_{res}^{+}=\frac{\lambda_{\text{res}\;}}{k_{B}\rho_{N}^{2/3}\sqrt{k_{B}T/m}}\times {\left(s^{+}\right)}^{2/3}.$$

The residual thermal conductivity is then obtained from the actual thermal conductivity at the temperature and density of interest, the dilute-gas thermal conductivity at the same temperature, and the critical enhancement of thermal conductivity by

(11) $$\lambda_{\text{res}}\left(T,\rho \right)=\lambda \left(T,\rho \right)-\lambda_{0}\left(T\right)-\Delta \lambda_{C}\left(T,\rho \right),$$

in which $\lambda_{0}(T)$ is the dilute-gas thermal conductivity at the temperature of interest and $\Delta \lambda_{C}(T,\rho )$ is the critical enhancement of thermal conductivity at the temperature and density of interest. The critical enhancement parameter is approximated using the Olchowy and Sengers crossover model [44] as follows:

(12) $$\Delta \lambda_{C}\left(T,\rho \right)=\frac{\rho C_{p}R_{D}k_{B}T}{6\pi \eta \varphi }\left(\overset{‾}{\Omega }-{\overset{‾}{\Omega }}_{0}\right),$$

(13) $$\overset{‾}{\Omega }=\frac{2}{\pi }\left[\left(\frac{C_{p}-C_{v}}{C_{p}}\right)arctan\left(q_{D}\varphi \right)+\frac{C_{v}}{C_{p}}q_{D}\varphi \right],$$

(14) $${\overset{‾}{\Omega }}_{0}=\frac{2}{\pi }\left[1-exp\left(\frac{-1}{{\left(q_{D}\varphi \right)}^{-1}+{\left(q_{D}\varphi \rho_{c}/\rho \right)}^{2}/3}\right)\right],$$

(15) $$\varphi =\varphi_{0}{\left(\frac{P_{c}\rho }{\Gamma \rho_{c}^{2}}\right)}^{v/\gamma }{\left[{\left.\frac{\partial \rho (T,P)}{\partial P}\right|}_{T}-{\left.\left(\frac{T_{ref}}{T}\right)\frac{\partial \rho \left(T_{ref},p\right)}{\partial p}\right|}_{T}\right]}^{v/\gamma },$$

in which $C_{p}$ is the isobaric heat capacity, $C_{v}$ is the isochoric heat capacity, $\rho_{c}$ is the critical molar density, and $\rho_{c}$ is the critical pressure. The aforementioned thermodynamic properties are estimated using the vtPR model from Sect. 3.2.1 implemented in REFPROP [38]. $R_{D},\;\nu$, and γ are universal parameters [40]. $\Gamma ,\;\phi ,\;T_{\text{ref}}$, and $q_{D}$ are fluid-specific parameters calculated using the methods outlined elsewhere [40, 45], and η is the viscosity. Limited literature data are available on viscosities of R-1130(E), hence we calculate the viscosity of R-1130(E) using the residual entropy scaling approach of Yang and coworkers [46] as outlined in the SI. The data presented in this study are well below the critical regime and the critical enhancement parameter is close to zero for all state points presented here. However, we still include the thermal conductivity critical enhancement formulation for completeness.

The dilute-gas thermal conductivity in Eq. 11 is calculated using the approximations detailed by Bell and coworkers [47] and Chichester and Huber [45] in which a Eucken approach deconstructs the thermal conductivity to the sum of translational modes and total internal degrees of freedom:

(16) $$\lambda =\lambda_{tr}+\lambda_{int}.$$

The contribution of the translational modes, $\lambda_{\text{tr}}$, can be approximated as

(17) $$\lambda_{tr}=\eta_{0}f_{tr}c_{v,tr}^{\left(0\right)},$$

in which $\eta_{0}$ is the dilute-gas viscosity in Pa·s approximated using

(18) $$\eta_{0}\left(T\right)=\frac{5}{16}\sqrt{\frac{mk_{B}T}{\pi }}\frac{1}{\sigma^{2}\Omega^{(2,2{)}^{*}}},$$

in which the reduced collision integral, $\Omega^{(2,2{)}^{*}}$, is estimated using the empirical formulation of Neufeld et al. [48]:

(19) $$\begin{array}{l} \Omega^{(2,2{)}^{*}}=1.16145T^{*(-0.14874)}+0.52487e^{-0.77320T*}+2.16178e^{-2.43787T*}\\ \;-0.0006435T^{*(0.14874)}sin(18.0323T^{*(-0.76830)}-7.27371) \end{array}.$$

With $T^{*}$ being the reduced temperature defined as $T^{*}=k_{B}T/\varepsilon .\;\sigma$ and ε are the L–J parameters of the fluid.

In Eq. 17, $f_{\text{tr}}$ equals 5/2 and the translational contribution to zero-density specific heat, $c_{v,tr}^{\left(0\right)}$, is set to 3R/(2M) in which R is the molar gas constant in J·mol^−1^·K^−1^ and M is the molar mass in kg·mol^−1^.

The second term in Eq. 16, the contribution due to the total internal degrees of freedom $\left(\lambda_{\text{int}}\right)$, is approximated using

(20) $$\lambda_{\text{int}}=\eta_{\rho \to 0}\left(\frac{\rho_{\text{mass}}D_{\text{self}}}{\eta_{\rho \to 0}}\right)c_{\text{int}}^{\left(0\right)},$$

with $\rho_{\text{mass}}D_{\text{self}}/\eta_{\rho \to 0}$, assumed to equal 1.32 as detailed elsewhere [47] and

(21) $$c_{\text{int}}^{\left(0\right)}=c_{v}^{\left(0\right)}-c_{v,\text{tr}}^{\left(0\right)},$$

in which $c_{v}^{\left(0\right)}$ is the constant-volume specific heat on a mass basis.

The plus-scaled residual thermal conductivity can then be approximated using

(22) $$\lambda_{\text{res}}^{+}=n_{1}\frac{s^{+}}{\xi }+n_{2}{\left(\frac{s^{+}}{\xi }\right)}^{1.5}+n_{3}{\left(\frac{s^{+}}{\xi }\right)}^{2}+n_{4}{\left(\frac{s^{+}}{\xi }\right)}^{2.5},$$

which, alongside the calculated zero-density thermal conductivity and the thermal conductivity critical enhancement, allows for approximation of the fluid thermal conductivity at the temperature and density of interest using Eqs. 7–11. ξ is a fluid-specific scaling factor and $n_{1},\;n_{2},\;n_{3}$, and $n_{4}$ are fitted parameters with global values presented by Yang and coworkers [26].

**Fig. 4:**
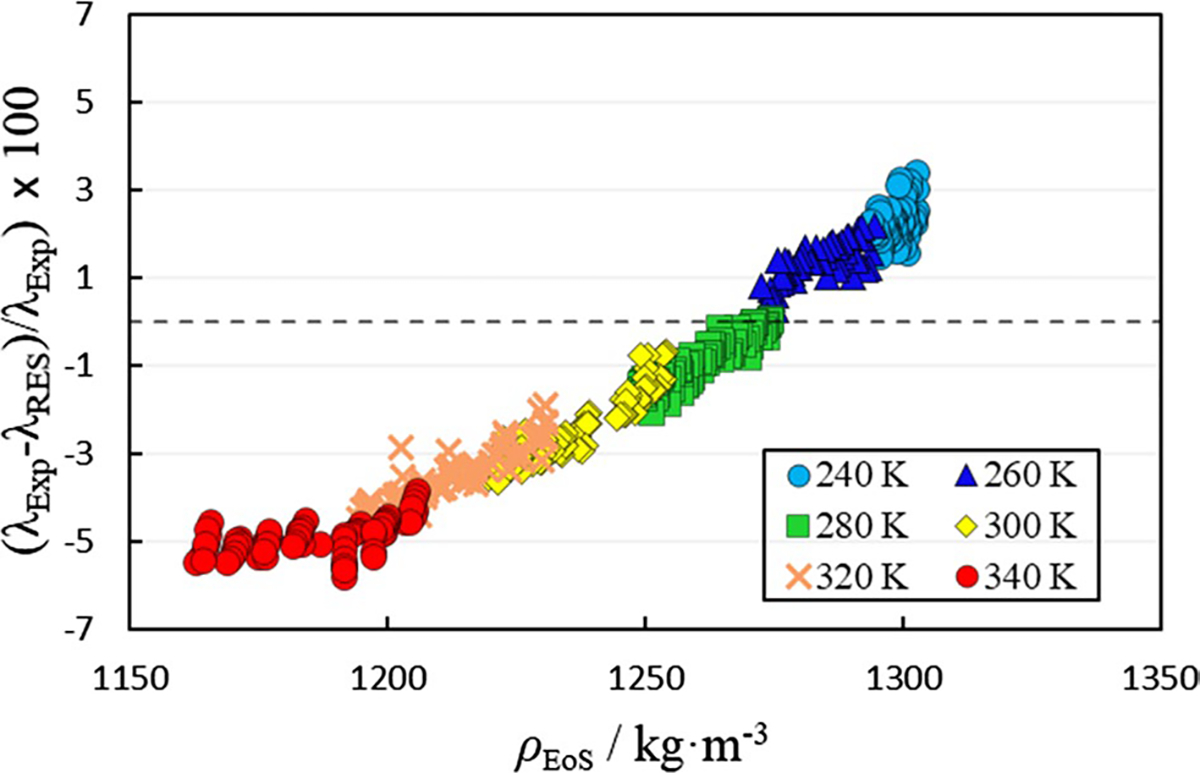
Comparison of experimental thermal conductivity values ($\lambda_{Exp}$) to thermal conductivity values calculated using the residual entropy scaling model with a scaling factor of 1.0 and global $n_{i}$ parameters from Table 2 ($\lambda_{\text{RES}}$) as a function of density ($\rho_{\text{EoS}}).\;\rho_{\text{EoS}}$ is the density calculated using the volume-translated Peng–Robinson EoS described in Sect. 3.2.1. Temperatures in legend are nominal temperatures

The first approach adopted in this study utilizes a scaling factor of 1.0 and global $n_{i}$ parameters. The global parameters are reproduced in Table 2. The zero-density thermal conductivity and the dimensionless residual thermal conductivity values at each state point are reproduced in the SI. The extensive modeling values presented in the SI serve as check values intended to aid the reader in implementing the model. Figure 4 illustrates the deviation of the thermal conductivity calculated using the RES model from the experimental values.

As illustrated in Fig. 4 and Table 2, utilizing a scaling factor of 1.0 and global $n_{i}$ parameters, the thermal conductivity can be approximated with an average absolute relative deviation (Δ_AARD_) of 2.55%, although with a significant systematic trend.

**Fig. 5:**
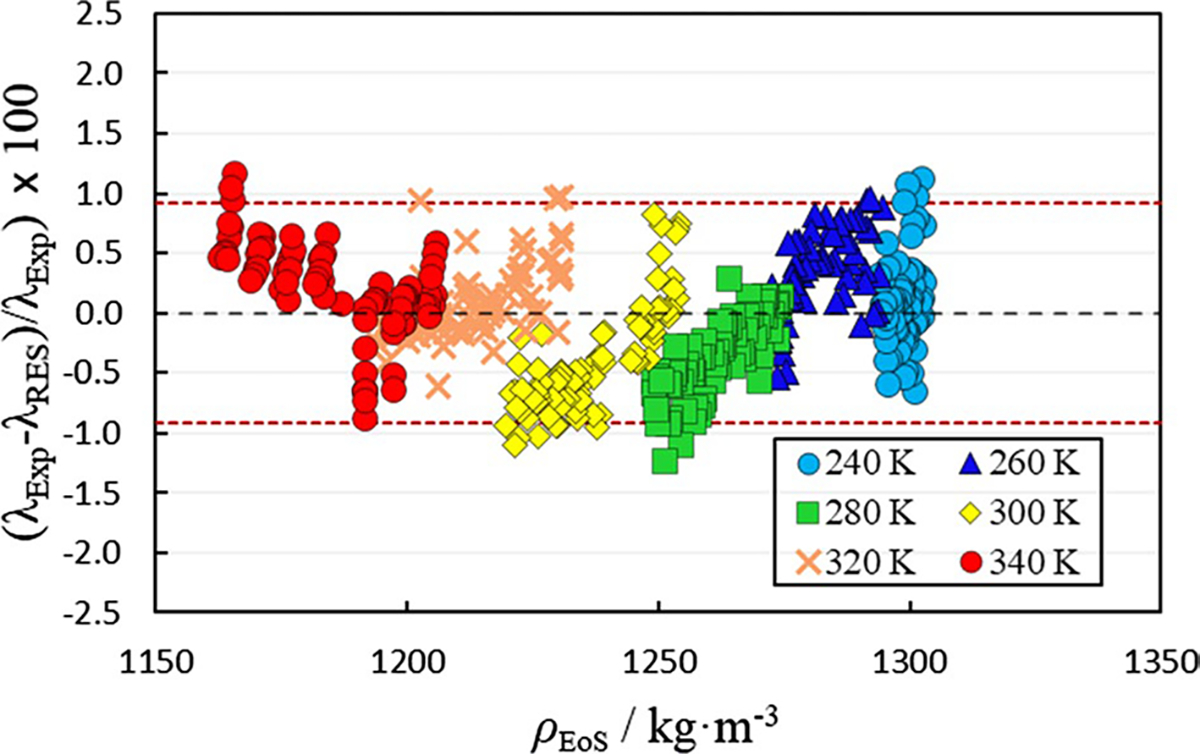
Comparison of experimental thermal conductivity values ($\lambda_{Exp}$) to thermal conductivity value calculated using the residual entropy scaling model with a scaling factor of 1.0 and fitted $n_{1}$ and $n_{3}$ parameters from Table 2 ($\lambda_{\text{RES}}$) as a function of density $\left(\rho_{\text{EoS}}\right).\;\rho_{\text{EoS}}$ is the density calculated using the volume-translated Peng–Robinson EoS described in sect. 3.2.1. Temperatures in legend are nominal temperatures

The RES approximations can be further improved by regressing different combinations of the $n_{i}$ parameters. Through regression of different combinations of parameters, it was concluded that the present system is most sensitive to $n_{1}$ and $n_{3}$ with either one not yielding more than a moderate improvement (~ 2.15% Δ_AARD_ compared to 2.55% Δ_AARD_ with global parameters) when fit individually. Using a generalized reduced gradient method that minimizes the Δ_AARD_ objective function, both the $n_{1}$ and $n_{3}$ parameters were regressed simultaneously yielding a Δ_AARD_ of 0.37% relative to the experimental data as illustrated in Fig. 5 and Table 2. The maximum deviation is improved to 1.23% and 95% of the data are represented within 0.92%.

Table 2 provides the parameters in the RES model for the two approaches to predicting/regressing the experimental thermal conductivity data with parameter values and Δ_AARD_ values for each respective approach. Setting the scaling factor to 1.0 and regressing all of the $n_{i}$ parameters to the experimental data results in the smallest Δ_AARD_ (0.32%) but is unnecessary as fitting only two parameters $\left(n_{1},n_{3}\right)$ results in comparable improvements (0.37% Δ_AARD_) relative to the use of the global parameters (2.55% Δ_AARD_). Nevertheless, the RES configuration with a default scaling factor of 1.0 and global parameters performs well, especially considering the combined expanded uncertainty of the experimental measurements.

## 4 Conclusions

Experimental thermal conductivity data of *trans*-1,2-dichloroethene (R-1130(E)) obtained using a dual-wire transient hot-wire instrument were reported at temperatures ranging from 240 K to 340 K and pressures up to 25 MPa. A total of 447 thermal conductivity data points were reported at six isotherms. Each isotherm includes data at nine pressure points, which were chosen to be at equal density increments starting at a pressure of 0.1 MPa (or slightly above the saturation pressure of R-1130(E) at temperatures above its normal boiling point) to a maximum pressure of 25 MPa. The reported experimental data have a combined expanded uncertainty of 1.4% at a 95% confidence interval. A transport property ECS model was used in a predictive mode and compared to the experimental data with an Δ_AARD_ of 6.89%. The measured data were then used to tune the ECS model parameters. The employed ECS with regressed parameters can reproduce the thermal conductivity to within the experimental uncertainty across the temperature and pressure regimes investigated. A RES method was also employed to predict thermal conductivity values. The RES method, using a default scaling factor of 1.0 and global parameters, models the experimental data exceptionally well with an Δ_AARD_ of 2.55% across the temperature and pressure regimes investigated. Regressing two empirical parameters to the experimental thermal conductivity data decreases the Δ_AARD_ to 0.37%, but like the ECS method with regressed parameters, this technique is a correlative approach. The presented data coupled with parameters of the ECS and RES models facilitate the calculation of thermal conductivity across a wide range of conditions.

## Supplementary Material

Supp1

## Figures and Tables

**Table 1:** Specifications of Chemicals

Chemical name	R-number	Chemical Structure	CAS no	Source	Purity^a^

*trafts*-1,2-Dichloroethene	R-1130(E)	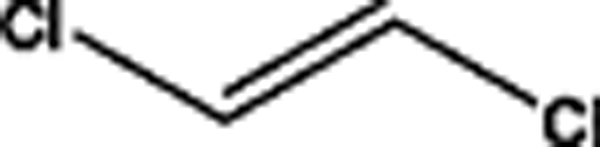	156-60-5	The Chemours Company^b^	0.991 mass fraction

a Samples degassed using freeze/pump/thaw method described in [28]. Purity assessed using ^1^H NMR

b Certain commercial equipment, instruments, or materials are identified in this paper in order to specify the experimental procedure adequately. Such identification is not intended to imply recommendation or endorsement by NIST, nor is it intended to imply that the materials or equipment identified are necessarily the best available for the purpose.

**Table 2:** Parameters and deviation of the residual entropy scaling model for thermal conductivity

ξ value	$n_{i}$	$n_{1}$	$n_{2}$	$n_{3}$	$n_{4}$	Δ_AARD_(%)

1.0	Global	3.636446	− 5.32826	4.543762	− 0.64335	2.55
1.0	$n_{1}$, $n_{3}$ fit	1.670547	− 5.32826	4.889275	− 0.64335	0.37

Global parameters, $n_{i}$, obtained from [26]. ξ: RES scaling factor

## Data Availability

The supplementary information includes the experimental thermal conductivity data and the uncertainties in variables, the residual entropy scaling model check values at each respective state point, the viscosity calculated using the extended corresponding states model and the residual entropy scaling model, performance assessment of the volume-translated Peng–Robinson EoS and the extended corresponding states model for equilibrium properties, and a (.FLD) fluid file for R-1130(E) that can be implemented in REFPROP v10.0. The NMR purity analysis methodology and technique are described in the SI. The experimental thermal conductivity data and associated variables are also presented in the NIST Public Data Repository (https://doi.org/https://doi.org/10.18434/mds2-3108).

## Footnotes

1 Commercial equipment, instruments, or materials are identified only to adequately specify certain procedures. Such identification does not imply recommendation or endorsement by the National Institute of Standards and Technology, nor does it imply that the identified products are necessarily the best available for the purpose.
