# Supplementary material for: Thermal Conductivity of Liquid trans-1,2-Dichloroethene (R-1130(E)): Measurement and Modeling
Source: Int J Thermophys. Author manuscript; Available in PMC 2025 Jan 14. (PMC11729616; doi:10.1007/s10765-024-03334-2)
Supplement: Supp1 [file NIHMS2022139-supplement-Supp1.zip › SI_R1130E_TCx_IJOT/SI_R1130E_TCx_IJOT/SI_R1130E_TCx_IJOT_FA.docx]

Supplementary Information

Thermal Conductivity of Liquid *trans*-1,2-Dichloroethene (R-1130(E)): Measurement and Correlation

Karim S. Al-Barghouti^1^, Aaron J. Rowane^2*^, Ian H. Bell^2^, Marcia L. Huber^2^, and Richard A. Perkins^2^

^1^ *Department of Chemical and Petroleum Engineering, University of Kansas, Lawrence, Kansas 66045, United States*

^2^ *Applied Chemicals and Materials Division, Material Measurement Laboratory, National Institute of Standards and Technology, Boulder, Colorado 80305, United States*

* Corresponding author Email: Aaron.Rowane@nist.gov

Contents contained in Supplementary Information

- Table of variables and uncertainties
- Performance of vtPR and ECS models for equilibrium properties described in section 3.2 of main text
- Transport property models for viscosity
- Measured thermal conductivity data
- Dimensionless residual thermal conductivity and zero-density thermal conductivity for RES model
- R-1130(E) fluid file (R1130E.FLD) compatible with the REFPROP v10 [1] computer program for EoS and ECS models.
- NMR sample purity analysis

**S.1. Variables and Uncertainties**

**Table S1**. Variables and uncertainties including relative uncertainties.

| **Variable / Units (Description)** | **Uncertainty** |
| --- | --- |
| *p* / MPa (measured pressure) | 7 kPa (coverage factor of *k*=2) |
| *q* / W·m^-1^ (heating power per unit length of wire) | 0.001 *i.e.* 0.1% (relative expanded uncertainty with coverage factor of *k*=2) |
| *T*_e_ / K (temperature of measurement i.e. wire temperature) | 20 mK (coverage factor of *k*=2) |
| STAT (expanded relative uncertainty of the slope of line of temp rise versus ln(*t*) relative to ideal temp rise versus ln(*t*)) | 0.001 *i.e.* 0.1% (relative expanded uncertainty with coverage factor *k*=2) |
| *T*_i_ / K (temperature of cell) | 5 mK (coverage factor of *k*=2) |
| *ρ*_EoS_ / kg·m^-3^ (density calculated using EoS described in section 3.2 of main text) | 0.003 *i.e.* 0.3% (relative expanded uncertainty with coverage factor of *k*=2) |
| λ / mW·m^-1^·K^-1^ (measured thermal conductivity) | 0.014 *i.e.* 1.4% (relative expanded uncertainty with coverage factor *k*=2) |

**S.2. EoS and ECS Modeling for Equilibrium Properties**

**S.2.1. Performance of Employed EoS Described in 3.2.1**

Comparisons of the results of the volume-translated Peng-Robinson equation (vtPR) and the extended corresponding states (ECS) model of Teraishi et al. [2] as implemented to R-1130(E) by Tanaka et al. [3] with the experimental data of Tanaka et al. [3] are presented in Figures S1 – S6. Figures S1 and S2 show comparisons with the vtPR and ECS models, respectively, for liquid phase experimental densities by Tanaka et al. [3] As discussed earlier, the estimated uncertainty of the experimental data is 0.3% at the 95.4% confidence level. Figure S1 shows the data are represented to within this level of uncertainty and systematic deviations are not observed for the vtPR model.


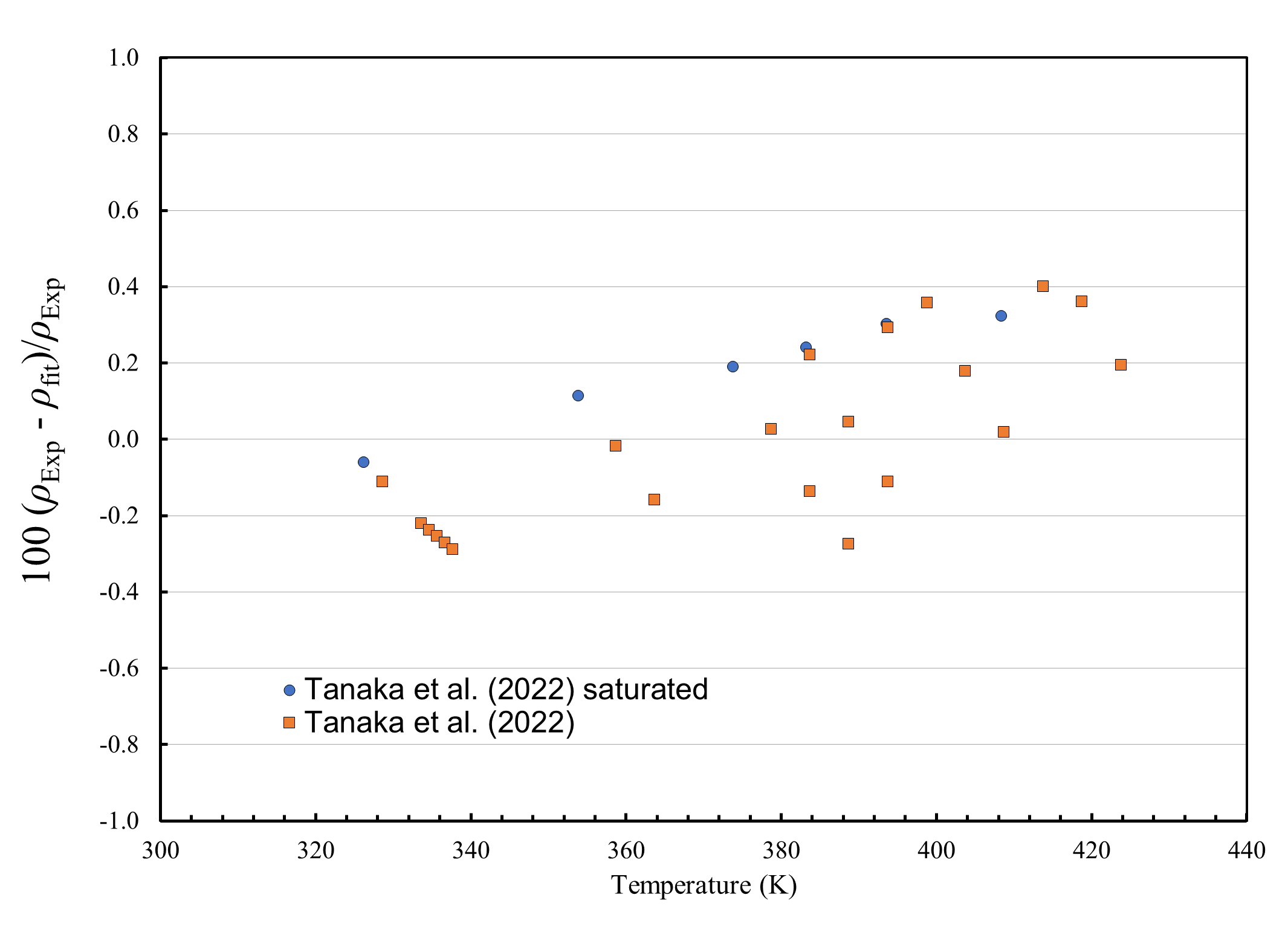


**Figure S1**. Comparisons of liquid density, vtPR model and data of Tanaka et al. [3]


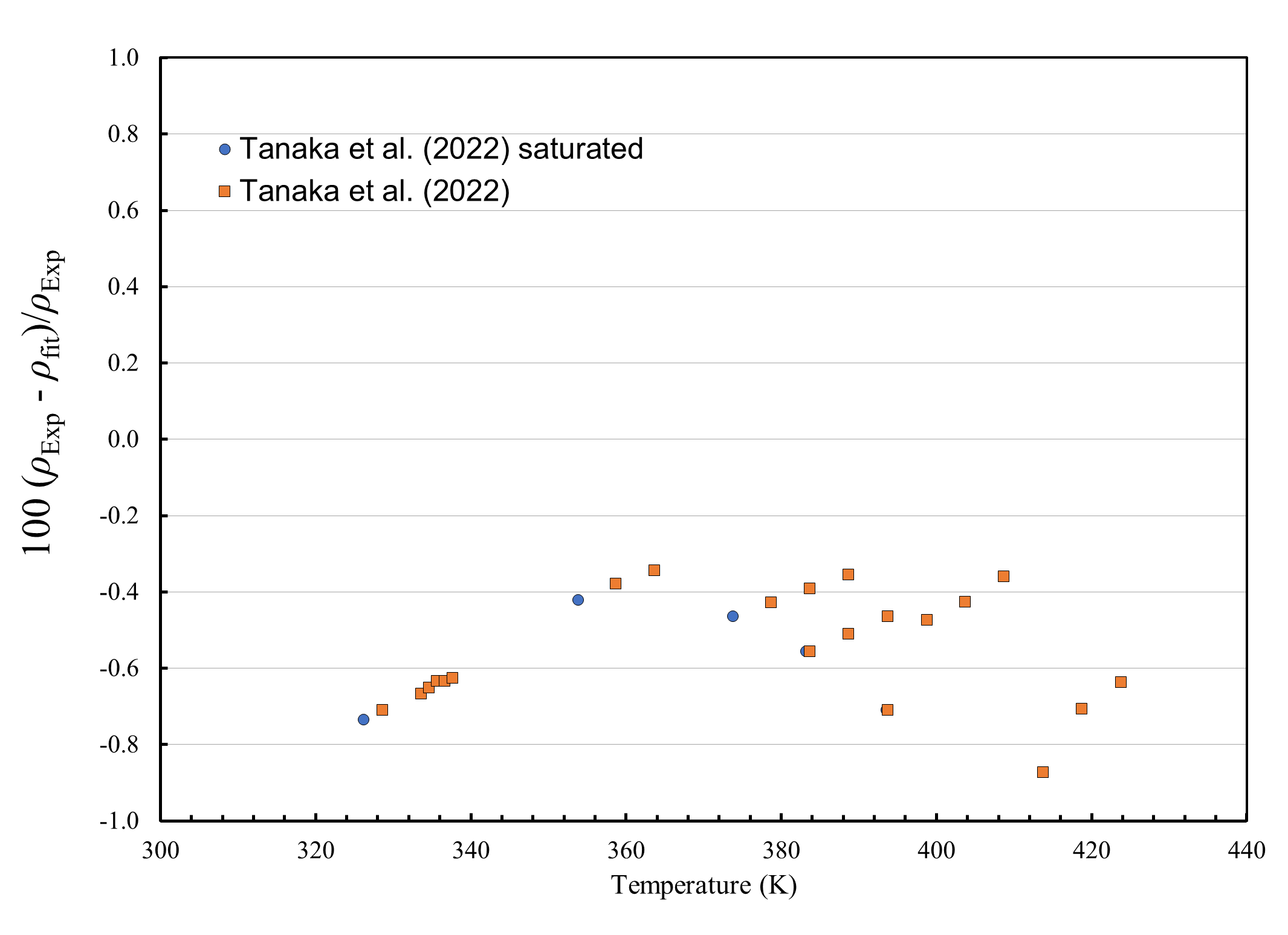


**Figure S2**. Comparisons of liquid density, ECS model [3].

Figures S3 (vtPR) and S4 (ECS) show comparisons of models with vapor phase experimental densities reported by Tanaka et al.[3] The estimated uncertainty of the vapor-phase experimental data is also 0.3% at the 95.4% confidence level. The volume-translated Peng-Robinson as well as the ECS model of Tanaka et al.[3] show comparable results with deviations of up to -4% and do not represent the experimental data to within the experimental uncertainty.


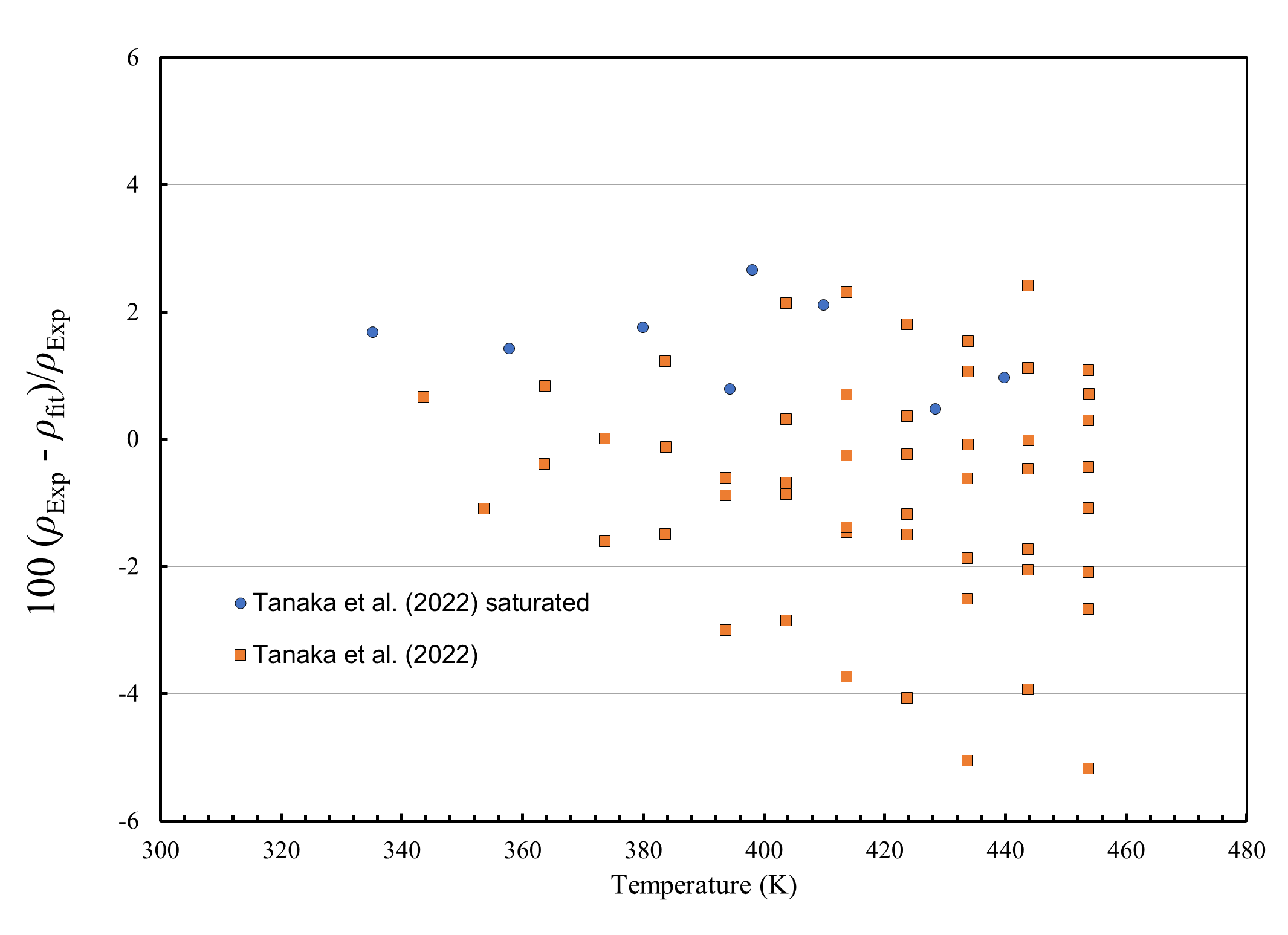


**Figure S3**. Comparisons of vapor density, vtPR model.


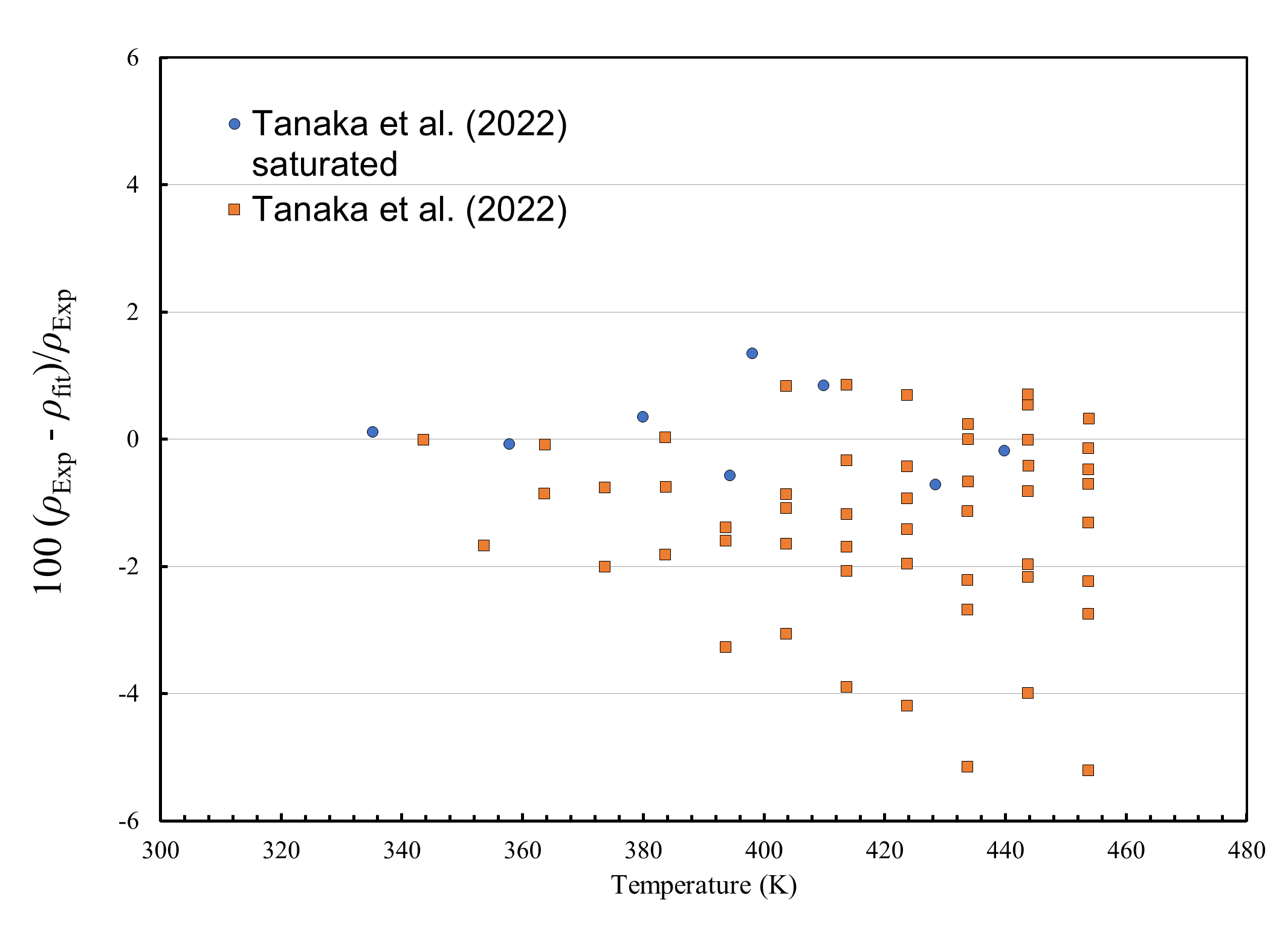


**Figure S4**. Comparisons of vapor density, ECS model[3].

Finally, figures S5 and S6 show the deviations of the vtPR and ECS model, respectively, in comparison to the vapor pressure data of Tanaka et al.[3] The experimental uncertainty is given as 0.3%. The ECS model gives slightly better results but neither of the models can represent the data to within experimental uncertainty. In the future we plan to develop an accurate Helmholtz equation of state when sufficient data become available. The models and fluid file presented here should be considered preliminary and should not be considered as a reference quality equation for R-1130(E).


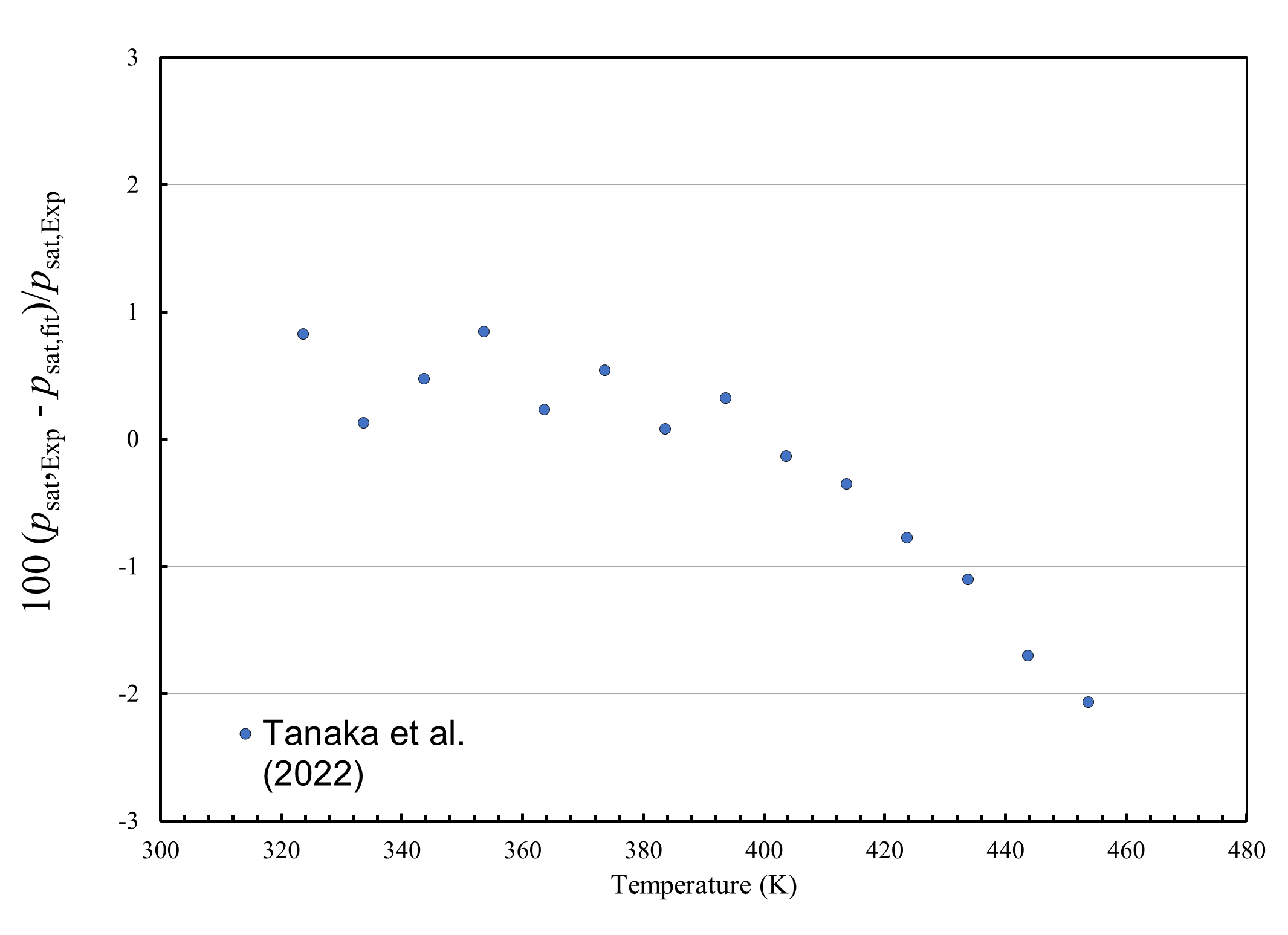


**Figure S5**. Comparisons of vapor pressure, vtPR model.


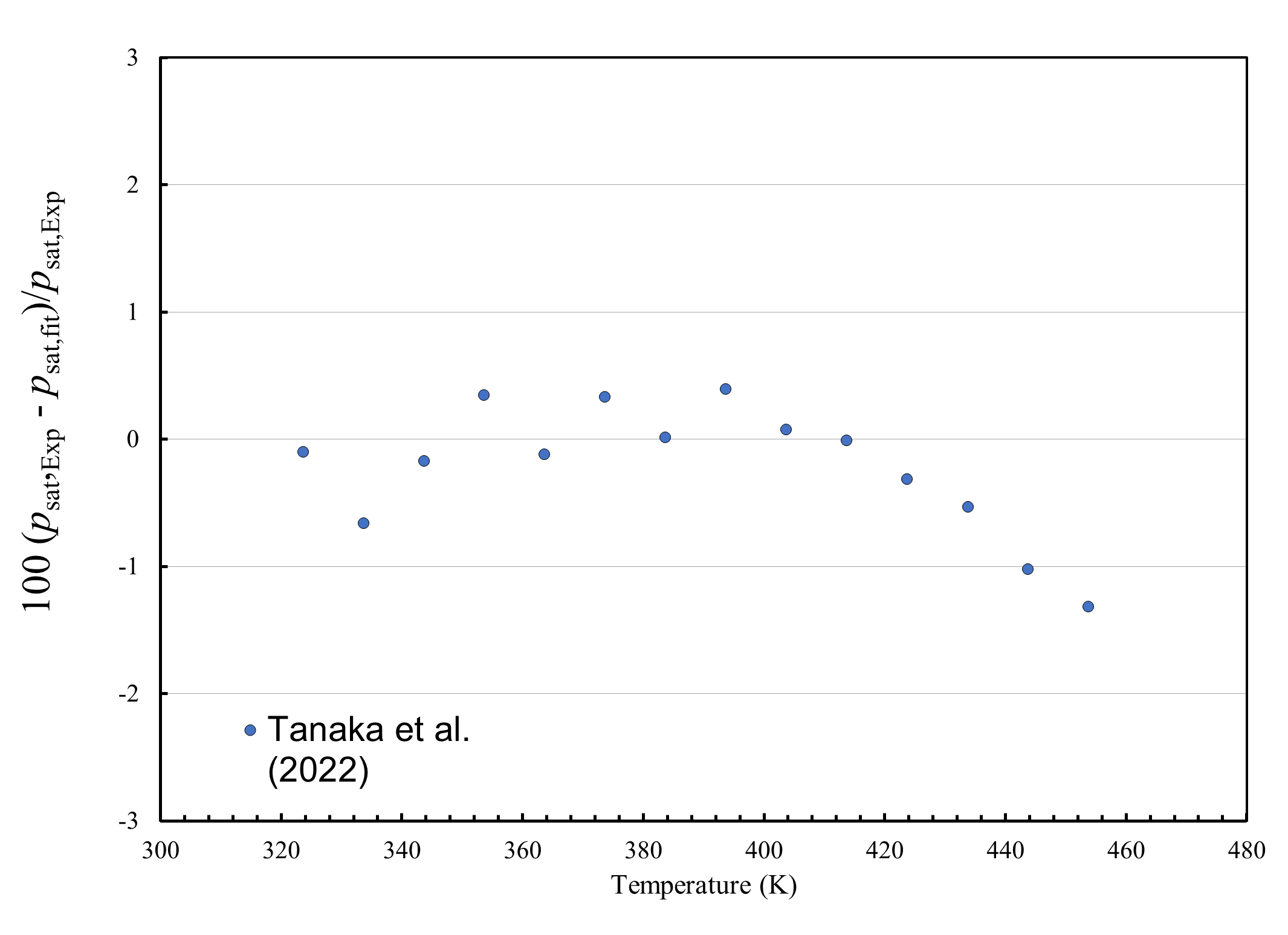


**Figure S6**. Comparisons of vapor pressure, ECS model.

**S.2.2 Transport Property Models for Viscosity**

**S.2.2.1 Extended corresponding states (ECS) Model for Viscosity**

The coefficients *c_k_* (Eq. 11 in ref.[4]) that are used to empirically correct the ECS viscosity

$\psi(\rho_{\text{r}})=\sum_{k=0}^{n} c_{k}\rho_{\text{r}}^{k}$ (S1)

were determined by fitting the experimental data of Awbery and Griffiths[5] and Ketelaar et al.[6] to a linear function in reduced density (*ρ*_r_ = *ρ*/*ρ*_c_ ), resulting in parameter values of *c*_0_ = 0.997079 and *c*_1_ = -0.0161478. The reference fluid used was R-134a. The results are shown in Figure S7. All viscosities are in the liquid phase at atmospheric pressure, no vapor data were found. The data are represented to within 4% with an average absolute relative deviation (Δ_AARD_) of 1.6%.


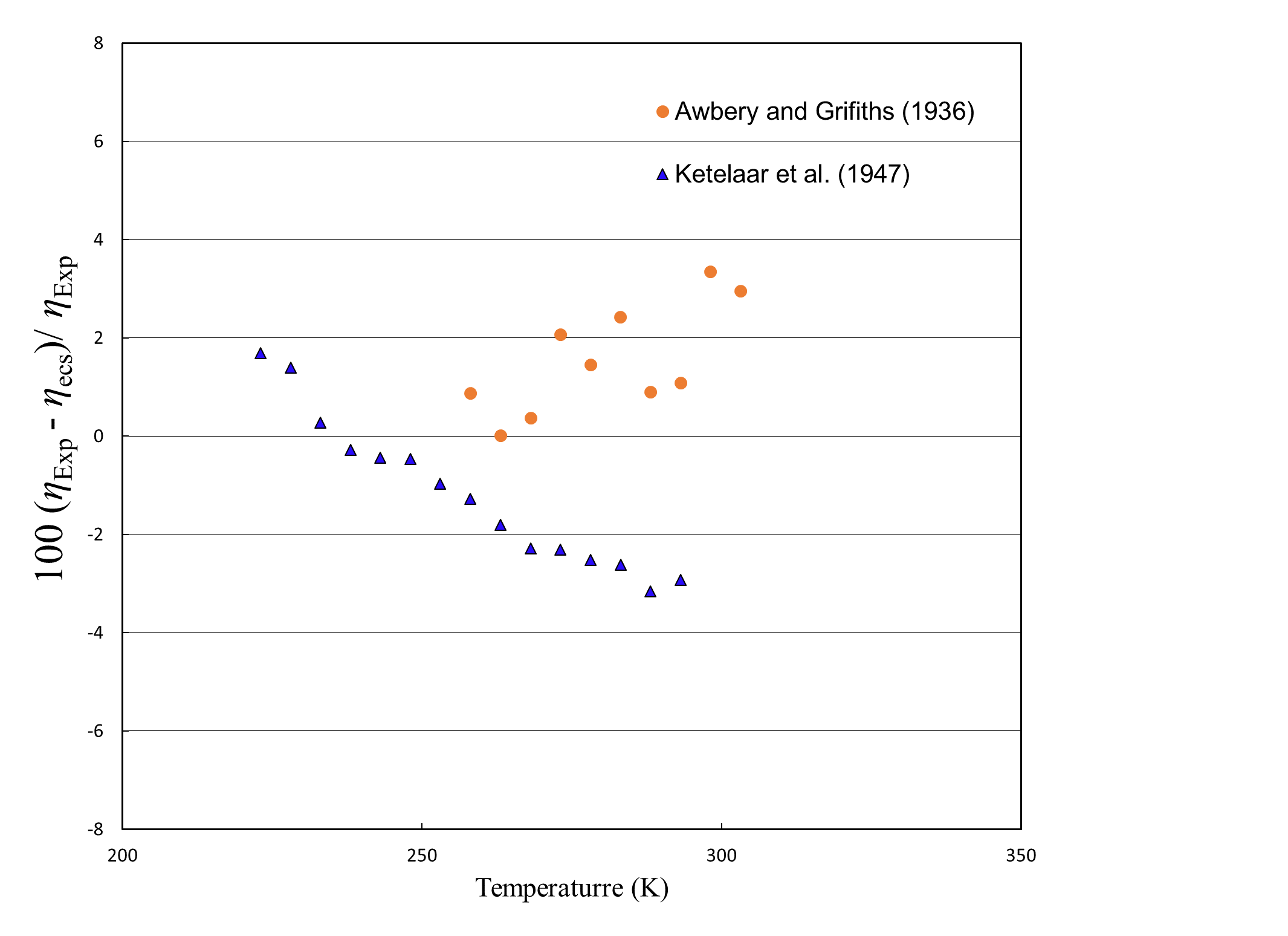


**Figure S7**. Comparisons of viscosity

**S.2.2.2 Residual Entropy Scaling (RES) Model for Viscosity**

The viscosity is approximated using the residual entropy scaling approach of Yang and coworkers [7] as follows

$\eta_{\text{res}}(T,\rho)=\eta(T,\rho)-\eta_{0}(T)$ (S2)

$\eta_{\text{res}}^{\text{+}}=\frac{\eta_{\text{res}}}{\rho_{\text{N}}^{2/3}\sqrt{mk_{\text{B}}T}}\times(s^{+})^{2/3}$ (S3)

In which *η*_res_ is the residual entropy at the temperature and density of interest, $\eta_{0}(T)$ is the dilute-gas viscosity obtained using correlations presented in the main text, and$\eta_{\text{res}}^{+}$is the plus-scaled residual viscosity.

The plus-scaled residual viscosity is calculated as

$\ln(\eta_{\text{res}}^{+}+1)=j_{1}\frac{s^{+}}{\xi}+j_{2}\left(\frac{s^{+}}{\xi}\right)^{1.5}+j_{3}\left(\frac{s^{+}}{\xi}\right)^{2}+j_{4}\left(\frac{s^{+}}{\xi}\right)^{2.5}$ (S4)

in which *j*_1_, *j*_2_, *j*_3_, and *j*_4_ are global parameters and ξ is a fluid-specific parameter. Here, we fit ξ to the available literature data [5, 6]. The values of the global parameters and ξ are reported in Table S2 alongside the percent average absolute deviation (Δ_AARD_) relative to experimental data at ambient pressure and temperatures from 243.15 K to 303.15 K. The largest observed deviation is -9.29% relative to experimental data.

| **Table S2**. Global fitted parameters and fluid-specific scaling factor (ξ) for RES viscosity model | | | | | |
| --- | --- | --- | --- | --- | --- |
| ***j*_1_** | ***j*_2_** | ***j*_3_** | ***j*_4_** | **ξ** | Δ_AARD_ |
| -0.448046 | 1.012681 | -0.381869 | 0.054674 | 1.05257 | 2.92% |
| Global parameters obtained from Ref. [7]. | | | | |  |

**S.3. Measured Thermal Conductivity**

The measured thermal conductivities, the temperature of the wire corresponding to each measurement, the measured pressure, the density calculated using the EoS, the power input, the temperature of the cell, the expanded combined uncertainty at a 95% confidence interval at each measurement point, and the deviation from the slope of the ideal temperature rise versus ln(*t*) line are reproduced in the “.txt” file provided.

**S.4. RES model dimensionless residual λ and zero-density λ**

Check values for the residual entropy scaling model and contributions for thermal conductivity are presented in the “.txt” file provided.

**S.5. NMR Sample Purity Analysis**

A degassed sample of R1130(E) (trans-1,2-dichloroethene) was presented in a stainless-steel sample cylinder (Swagelok 304L-HDF4-2250). To prepare an NMR sample of neat R-1130(E), a simple manifold with a central T was assembled. The glass NMR tube and the sample cylinder were connected opposite of each other on the central T. The vacuum line was connected to the third position on the T (Fig. S8). The manifold and the glass sample tube were evacuated for about 30 minutes while the fluid in the sample cylinder was warmed with a heat lamp. At this point, the sample cylinder was pleasantly warm to the touch (about 35 °C). Then the bottom half of the glass NMR tube was immersed in liquid nitrogen, the valve to the vacuum line was closed, and the valve on the sample cylinder was opened. Within a few seconds, the glass sample tube was about half full of liquid, so the valve on the sample cylinder was closed. Then the PTFE valve on the top of the glass NMR tube was closed and the NMR tube was removed from the manifold. The NMR tube was immediately transferred into the benchtop NMR and a cardboard box was placed over the sample bore to exclude ambient light. The sample experienced less than 5 minutes of ambient light exposure during this process.

The NMR sample went through a 5 min shimming routine, after which the linewidth (at 50 %) was 0.709 Hz. Test spectra for ^1^H and ^13^C were collected (about 15 minutes total). Then, a ^1^H spectrum was collected by averaging 1064 scans (90° pulse, acquisition time of 3.2 s, 16k data points, and pulse repetition time of 4.0 s). This spectrum took about an hour to collect. The spectrum was phased manually, after which automated baseline flattening was applied. Line broadening of 0.2 Hz resulted in a signal to noise ratio of 6.1 × 10^3^ for the main sample peak.

The spectrum of R-1130(E) consists of a singlet peak at 6.33 ppm with characteristic carbon satellites (*J*_C-H_ ~200 Hz, each satellite is a doublet). The spectrum was referenced to trace acetone (2.05 ppm) in a separately prepared sample (see below). A multitude of impurity peaks appeared in the range of 0.7 ppm to 3.0 ppm. The total intensity of the impurity peaks was 0.9 %. **Thus, the sample of R-1130(E) is (99.10 ± 0.10) % pure by ^1^H NMR.**

There were two general concerns about this purity analysis. First, this sample has some chemical instability, and it is sensitive to light. The question was, did the impurities come from the stainless-steel sample container or did they form during the sample handling and analysis. Two additional spectra were collected to check sample stability. A second spectrum was collected after the sample had been in the spectrometer for 4 days. Then the sample was removed from the spectrometer and left on the benchtop in 2-1107 (with all the lights on) for 30 minutes before a third spectrum was collected. The second and third spectra showed purities of 99.25 % and 99.22%, respectively. Thus, sample decomposition was ruled out. The second concern was that the impurity peaks looked suspiciously like a hydrocarbon oil. The question was, did they come from the sample itself or did they come from the transfer line between the stainless-steel sample container and the glass NMR tube. The transfer line and the NMR sample cell were rinsed with acetone before taking a second sample from the stainless-steel sample container. A ^1^H spectrum of this sample showed a purity of 99.17 % with the same suite of impurities as the first sample. This spectrum also had a small acetone peak (excluded from the purity value listed above), which was used to reference the spectral peaks. In any case, the lack of a change in the spectrum after rinsing the transfer line and NMR tube suggests that the contamination did not occur during sample preparation.


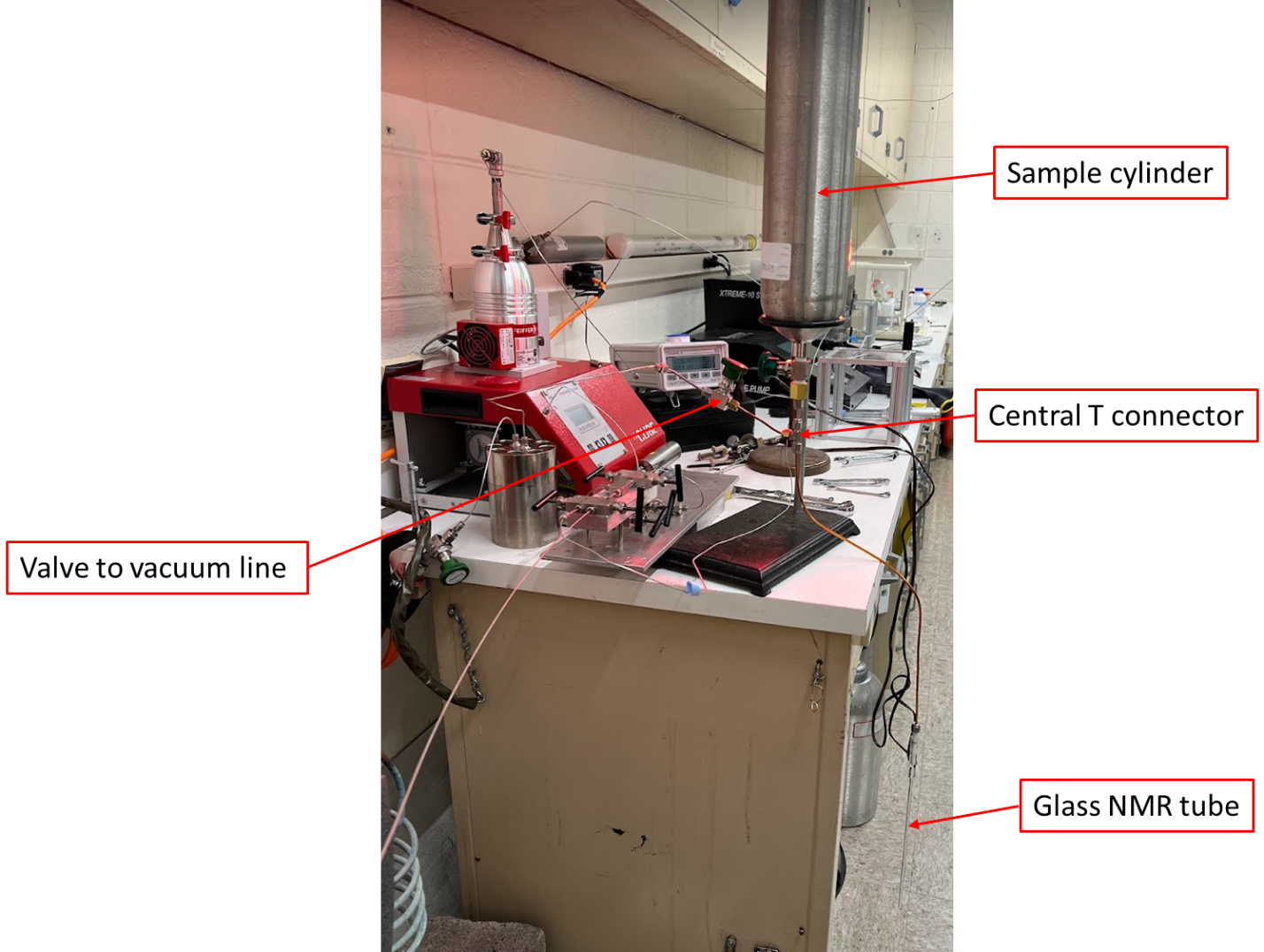


**Figure S8**. NMR filling setup.

**References**

1. E.W. Lemmon, I.H. Bell, M.L. Huber, M.O. McLinden, NIST Standard Reference Database 23: Reference Fluid Thermodynamic and Transport Properties-REFPROP, Version 10.0, National Institute of Standards and Technology, Standard Reference Data Program, Gaithersburg, 2018, doi:10.18434/T4/1502528

2. R. Teraishi, Y. Kayukawa, R. Akasaka, K. Saito, Int. J. Refrig. 131, 33 (2021) doi:10.1016/j.ijrefrig.2021.08.013

3. K. Tanaka, C. Kondou, S. Fukuda, R. Akasaka, Int. J. Thermophys. 43 (5), 69 (2022) doi:10.1007/s10765-022-02986-2

4. M.L. Huber, Models for Viscosity, Thermal Conductivity, and Surface Tension of Selected Pure Fluids as Implemented in REFPROP v10.0. NIST Interagency/Internal Report (NISTIR) 2018, No. 8209, doi:10.6028/NIST.IR.8209

5. J.H. Awbery, E. Griffiths, Proc. Phys. Soc. 48, 372 (1936) doi:10.1088/0959-5309/48/3/303

6. J.A.A. Ketelaar, L. Devries, P.F. Vanvelden, J.S. Kooy, Recl. Trav. Chim. Pays-Bas 66 (12), 733 (1947)

7. X. Yang, X. Xiao, E.F. May, I.H. Bell, J. Chem. Eng. Data 66 (3), 1385 (2021) doi:10.1021/acs.jced.0c01009
